# Relationship between movement time and hip moment impulse in the sagittal plane during sit-to-stand movement: a combined experimental and computer simulation study

**DOI:** 10.1186/s12938-018-0486-4

**Published:** 2018-04-27

**Authors:** Takuma Inai, Tomoya Takabayashi, Mutsuaki Edama, Masayoshi Kubo

**Affiliations:** 1 Institute for Human Movement and Medical Sciences, Niigata University of Health and Welfare, 1398 Shimami-cho, Kita-ku, Niigata, Niigata 950-3198 Japan; 2 Department of Rehabilitation, Oguma Orthopedics Clinic, 5-8-9 Koshin, Nishi-ku, Niigata, Niigata 950-2023 Japan

**Keywords:** Sit-to-stand movement, Hip joint, Impulse, Moment, Time

## Abstract

**Background:**

The association between repetitive hip moment impulse and the progression of hip osteoarthritis is a recently recognized area of study. A sit-to-stand movement is essential for daily life and requires hip extension moment. Although a change in the sit-to-stand movement time may influence the hip moment impulse in the sagittal plane, this effect has not been examined. The purpose of this study was to clarify the relationship between sit-to-stand movement time and hip moment impulse in the sagittal plane.

**Methods:**

Twenty subjects performed the sit-to-stand movement at a self-selected natural speed. The hip, knee, and ankle joint angles obtained from experimental trials were used to perform two computer simulations. In the first simulation, the actual sit-to-stand movement time obtained from the experiment was entered. In the second simulation, sit-to-stand movement times ranging from 0.5 to 4.0 s at intervals of 0.25 s were entered. Hip joint moments and hip moment impulses in the sagittal plane during sit-to-stand movements were calculated for both computer simulations.

**Results and conclusions:**

The reliability of the simulation model was confirmed, as indicated by the similarities in the hip joint moment waveforms (*r* = 0.99) and the hip moment impulses in the sagittal plane between the first computer simulation and the experiment. In the second computer simulation, the hip moment impulse in the sagittal plane decreased with a decrease in the sit-to-stand movement time, although the peak hip extension moment increased with a decrease in the movement time. These findings clarify the association between the sit-to-stand movement time and hip moment impulse in the sagittal plane and may contribute to the prevention of the progression of hip osteoarthritis.

## Background

Osteoarthritis is caused by excessive mechanical stress on articular cartilage [1], which leads to the development of osteophytes and joint-space narrowing [2]. This is associated with a decrease in functional ability [3], range of motion [4, 5], muscle strength [6, 7], and health-related quality of life [8]. Thus, preventing morbidity associated with the progression of hip osteoarthritis is essential. Some risk factors for hip osteoarthritis, such as age and sex, have been reported previously [9]. However, until recently, the biomechanical risk factors for hip osteoarthritis were unknown.

Recently, Tateuchi et al. [10] identified biomechanical risk factors related to joint-space narrowing in patients with hip osteoarthritis. They evaluated gait parameters at baseline using a three-dimensional motion capture system and examined the degree of hip joint space narrowing after 12 months. They proposed a new index called the daily cumulative hip moment, which is the product of the hip moment impulse during the stance phase and mean steps per day, and demonstrated that high daily cumulative hip moments in the frontal and sagittal planes were risk factors for hip osteoarthritis. We consider that repeated hip moment impulses in gait and other movements in daily life may encourage the progression of hip osteoarthritis. The daily cumulative hip moments calculated by Tateuchi et al. [10] were based on walking alone. The evaluation of hip moment impulse as an index of hip joint load during various movements (e.g., stairs, sit-to-stand movement, and sloped walking) in daily life may be important in identifying movement patterns with a low hip moment impulse.

Sit-to-stand movements are necessary in daily life and are performed approximately 60 times per day [11]. A large hip extension moment is required for the sit-to-stand movement [12–16]. Repeated sit-to-stand movements may cause repetitive hip moment impulses in the sagittal plane. Therefore, we believe that it is important to identify sit-to-stand movement patterns with low hip moment impulses in the sagittal plane and teach such movements to patients with hip osteoarthritis.

A simple way to modify the hip moment impulse in the sagittal plane during sit-to-stand movement is to change the movement time (i.e., change the movement speed). Yoshioka et al. [14] reported that a short sit-to-stand movement time (i.e., a fast sit-to-stand movement) caused a high peak hip extension moment. They found that the sum of the peak hip and knee joint moments increased exponentially when the total sit-to-stand movement time was below 2–3 s (i.e., time from seat-off to standing posture was below 1.12–1.68 s [= 56% × 2–3 s] [14]). The hip moment impulse in the sagittal plane during the sit-to-stand movement is calculated by integrating the hip extension and flexion moments; therefore, the increase in peak hip extension moment by a fast sit-to-stand movement may cause an increase in hip moment impulse in the sagittal plane. However, the time for a fast sit-to-stand movement is short compared to the time required for a slow sit-to-stand movement. Since the integration time of a fast sit-to-stand movement is short compared to that of a slow sit-to-stand movement, the fast sit-to-movement may cause a decrease in hip moment impulse in the sagittal plane. Hence, it is unknown whether the change in sit-to-stand movement time increases or decreases the hip moment impulse in the sagittal plane.

An investigation of the relationship between the movement time and hip moment impulse during sit-to-stand movement may help to identify a movement pattern with a low hip moment impulse in the sagittal plane. Several studies [14, 17–25] have focused on changes in sit-to-stand movement time, and some studies [14, 18, 21–23] have examined the effect of sit-to-stand movement time on hip joint moment in the sagittal plane (or hip extension moment). However, these studies did not examine hip joint moment impulses during sit-to-stand movements. Furthermore, although other studies [17, 19, 20, 24, 25] set various sit-to-stand movement times as conditions, they focused on other indices (e.g., power [17], acceleration [17, 19], joint angle [20, 24], muscle activation [20], reaction time [24], and momentum of center of mass [25]). From these observations, the relationship between movement time and hip moment impulse in the sagittal plane during sit-to-stand movement is unknown.

Therefore, the purpose of this study is to examine the relationship between movement time and hip moment impulse in the sagittal plane during sit-to-stand movement. We hypothesized that the hip moment impulse in the sagittal plane during sit-to-stand movement decreases with a decrease in the movement time.

An experiment alone may be inadequate to achieve our objective since a change in the sit-to-stand movement time may result in altered kinematics [21, 26–29], which may affect the hip moment impulse in the sagittal plane. Therefore, we used a combination of an experiment and computer simulation to examine the effect of movement time on hip moment impulse in the sagittal plane.

## Methods

### Protocol

**Fig. 1:**
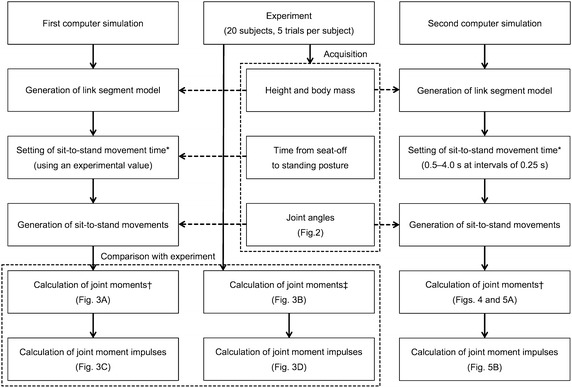
Study flowchart. *Sit-to-stand movement time of this study means the time from seat-off to the completion of the sit-to-stand movement. ^†^In the computer simulations, inverse dynamics were conducted from HAT to foot. ^‡^In the experiment, inverse dynamics were conducted from foot to HAT using the actual ground reaction force. *HAT* head, arm, and trunk

**Fig. 2:**
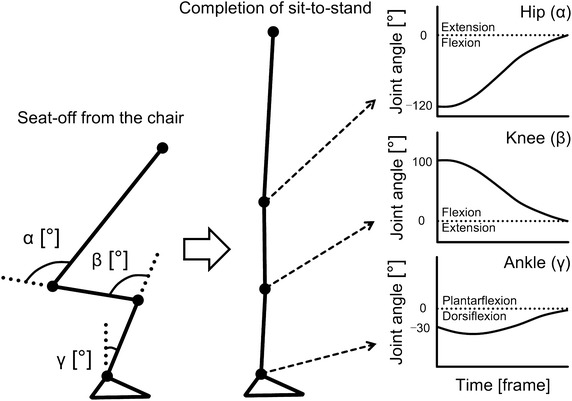
Calculation of joint angles during the sit-to-stand movement. The angles of the proximal segment relative to the distal segment during the sit-to-stand movement were calculated for each experiment trial. Counterclockwise was defined as plus, and clockwise was defined as minus

Figure 1 shows the study flowchart. First, we conducted an experiment to obtain normal sit-to-stand movement times in the study subjects. For the first and second computer simulations, we created a simulation model for sit-to-stand movement. The simulation model outputs the moment waveforms of the hip, knee, and ankle joints by using height, body mass, sit-to-stand movement time, and joint angles of sit-to-stand movement (Fig. 2). In the first computer simulation, we confirmed the similarity of the kinetics in the simulation model and the experiment. In the second computer simulation, we examined the hip moment impulse in the sagittal plane during various sit-to-stand movement times using the simulation model tested in the first computer simulation.

### Experiment and analysis

Twenty healthy subjects were recruited for the present study (5 men and 15 women; mean age, 18.8 ± 0.8 years; mean height, 1.65 ± 0.09 m; mean body mass, 53.9 ± 7.2 kg). The inclusion criteria were as follows: (1) no self-reported body pain, (2) no orthopedic and neurological disease, and (3) no surgeries. Informed consent was obtained in writing from all subjects prior to the study, which was approved by the ethics committee of the Niigata University of Health and Welfare (No. 17797–170412).

The subjects were asked to stand up with both arms on their chest at a natural self-selected speed from a chair measuring 0.4 m in height [14]. Five trials were performed for each subject. A three-dimensional motion capture system (Vicon, Oxford, UK) with 13 cameras was used to capture the sit-to-stand movements of each subject. Twenty-one reflective markers were attached on the bilateral acromion processes, anterior superior iliac spines, greater trochanters, lateral and medial epicondyles of the femur, lateral and medial malleoli, first and fifth metatarsal heads, heels, and at the midpoint of the posterior superior iliac spine of each subject. The sampling rate of the motion capture system was 100 Hz. Three force plates (AMTI, Watertown, MA) were used. The sampling rate of the force plates was 1000 Hz. The marker trajectories and ground reaction forces (right foot and both buttocks) were low-pass filtered using a fourth-order Butterworth filter with a cut-off frequency of 6 Hz.

We analyzed the right leg for each experimental sit-to-stand movement trial. The hip joint center was set using Bell’s method [30]. The knee joint center was set as the midpoint of the lateral and medial epicondyles of the femur and the ankle joint center was set as the midpoint of the lateral and medial malleoli. Body parameters (position of the center of mass, segment mass, and radius of gyration) were set based on a previous study [31]. The start time was defined as the time when the vertical ground reaction force of the buttocks was zero. The finish time was defined as the time when the vertical velocity of the right acromion was slower than 0.05 m/s.

A link segment model having four segments (right foot, right shank, right thigh, and HAT [head, arms, and trunk]) was used to analyze all experimental sit-to-stand movement trials. The hip, knee, and ankle joint moments during an experimental sit-to-stand movement for each subject were calculated based on inverse dynamics from foot to HAT (iterative Newton–Euler method [32]) using actual marker trajectories and ground reaction forces.

To calculate hip moment impulse in the sagittal plane during experimental sit-to-stand movement, anterior–posterior and superior-inferior components of ground reaction forces (i.e., two-dimensional components) were used. The hip, knee, and ankle joint moment impulses were calculated by integration of their respective joint moments during sit-to-stand movement as follows:

$${\text{I}}_{\text{j}} = \int\limits_{\text{t}_{0}}^{\text{T}} {{\text{M}}_{\text{j}} {\text{(t)dt}}}$$

I: joint moment impulse in the sagittal plane; j: hip, knee, or ankle joint; t_0_: timing of seat-off; T: timing of completion of sit-to-stand movement; and M: joint moment in the sagittal plane.

Additionally, the hip, knee, and ankle joint angles (Fig. 2) during all sit-to-stand movements (100 trials = 20 subjects × 5 trials) were calculated for use in the computer simulations.

### Computer simulation and analysis

We created the simulation model with a link segment model for the first and second computer simulations. Using the segment length ratios reported by Contini [33], we calculated the segment lengths of the link segment model using height. Further, using the ratios of the segment masses reported by de Leva [31], we calculated the segment masses of the link segment model using body mass. In the first and second computer simulations, the joint angles obtained from the experimental sit-to-stand movements were used to reproduce the same kinematics (Fig. 1).

In the first computer simulation, the movement times obtained from the experiment were used to calculate the joint moments and moment impulses in the sagittal plane of the hip, knee, and ankle joints during sit-to-stand movements (Fig. 1). Thus, 100 sit-to-stand movements (20 subjects × 5 trials) were analyzed in the first computer simulation. Similarities across the hip, knee, and ankle joint moment waveforms between the first computer simulation and the experiment were evaluated using Pearson’s correlation coefficients. Moreover, to confirm whether the joint moment waveforms of the first computer simulation were quantitatively reasonable compared to those of the experiment, we used “normalized integral error” [34]. In the second computer simulation, 15 patterns of sit-to-stand movement based on different times (0.5–4.0 s at intervals of 0.25 s) were entered for each sit-to-stand movement. Therefore, 1500 sit-to-stand movements (20 subjects × 5 trials × 15 sit-to-stand movement times) were analyzed in the second computer simulation.

To calculate the hip joint moment during a sit-to-stand movement in the second computer simulation, we first changed the sampling rate of joint angles obtained from the experiment trials from 100 to 1000 Hz. For example, when a sit-to-stand movement time in an experimental trial is 0.98 s, the number of frames of the joint angles during the sit-to-stand movement was changed from 98 frames (i.e., 100 Hz) to 980 frames (i.e., 1000 Hz) using a linear interpolation. After that, we changed the number of frames for a target sit-to-stand movement time (0.5–4.0 s at intervals of 0.25 s) using a linear interpolation for the second computer simulation. In the above example, to simulate a sit-to-stand movement of 0.75 s in the second computer simulation, the number of frames was changed from 980 to 750 frames. After this procedure, the inverse dynamics from HAT to foot were used (see [14] for detail procedure). Incidentally, velocities (or angular velocity) and accelerations (or angular acceleration) of center of masses were calculated using the central difference formula. Moreover, the hip moment impulse during the sit-to-stand movement was calculated by integrating the hip joint moment. All sit-to-stand movements in the two computer simulations were assumed to have bilateral symmetry.

We performed multiple comparisons to confirm whether the changes in sit-to-stand movement times affect hip moment impulses (or peak hip extension moments). Significance was assumed for *p* < 0.05, and all *p* values that were obtained using a paired t test or Wilcoxon signed-rank test were adjusted using Holm correction. All analyses of the sit-to-stand movements in the experiment and the two computer simulations were conducted using MATLAB (MathWorks, USA) and Scilab (Scilab Enterprises, France), and statistical analyses were conducted using R language (R Development Core Team).

## Results

Table 1 presents the comparison of parameters during normal sit-to-stand movements during the experiment, the first computer simulation, and previous studies.

**Table 1:** Comparison of parameters during normal sit-to-stand movement in the experiment, computer simulation, and previous studies

	Experiment, mean (SD)	First computer simulation	Previous studies
Sit-to-stand movement time^c^ (s)	0.73 (0.16)	–	0.74 (0.18)^a^
Hip flexion angle at seat-off (°)	92.0 (6.0)	–	93.4 (8.4)^b^
Peak hip extension moment (Nm/kg)	0.71 (0.16)	0.85 (0.17)	0.62 (0.12)^b^

*SD* standard deviation

^a^Yoshioka et al. [15]

^b^Doorenbosch et al. [12]

^c^Sit-to-stand movement time is the time from seat-off to the completion of the sit-to-stand movement

**Fig. 3:**
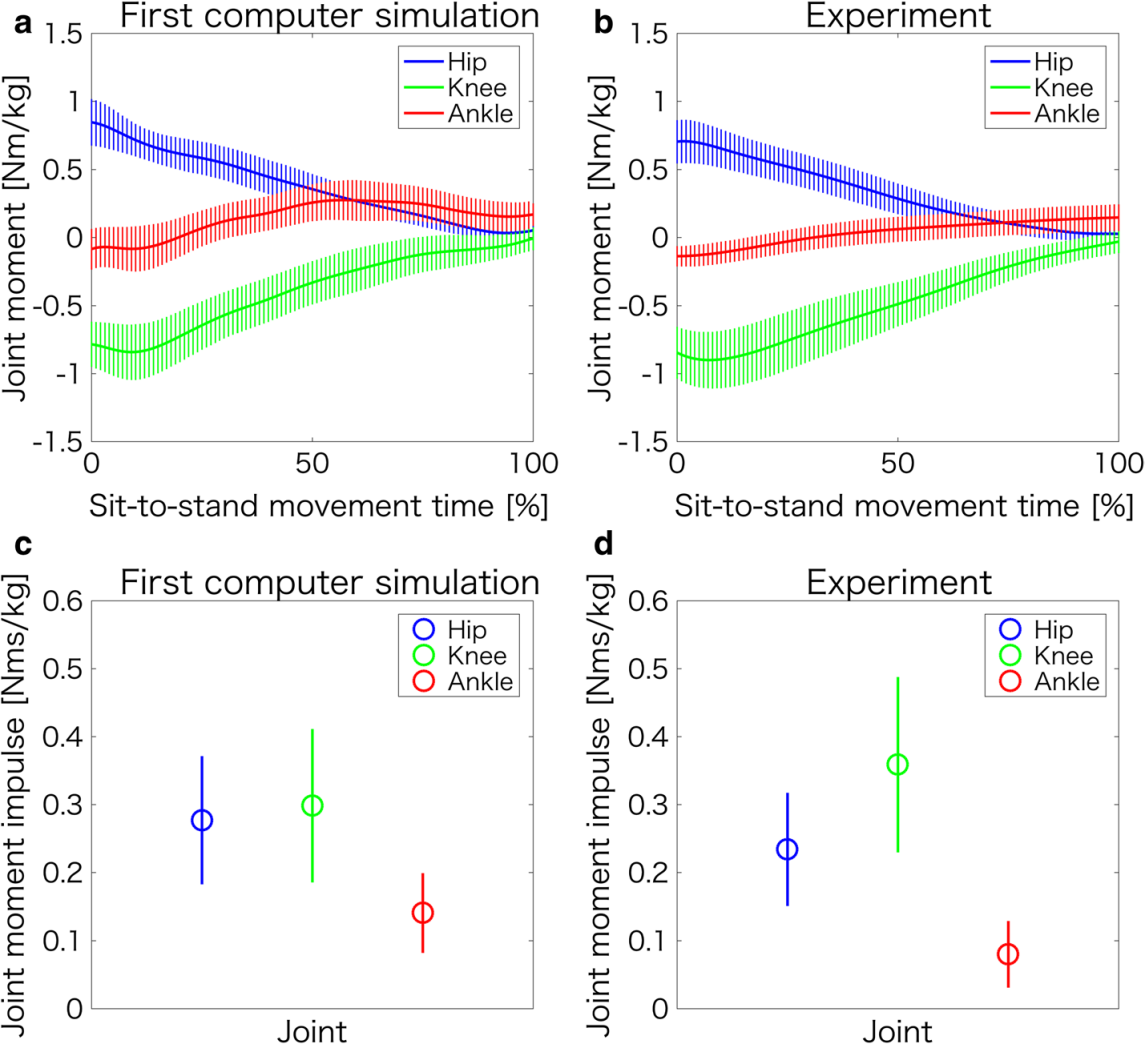
Joint moments and joint moment impulses during normal sit-to-stand movements in the first computer simulation and experiment. **a**, **c** Indicate the joint moments and the joint moment impulses in the first simulation, respectively, while **b**, **d** indicate the joint moments and the joint moment impulses in the experiment, respectively. In **a**, **b**, the positive values of the hip, knee, and ankle joint moments represent the hip extension moment, knee flexion moment, and ankle plantarflexion moment

Figure 3a–d demonstrate joint moments and joint moment impulses during normal sit-to-stand movement in the first computer simulation and in the experimental trials. In Fig. 3a (first computer simulation) and Fig. 3b (experiment), both waveforms for the hip, knee, and ankle joint moments were similar. The Pearson’s correlation of the hip, knee, and ankle joint waveforms between the first computer simulation and the experiment were 0.99 (*p* < 0.001), 0.98 (*p* < 0.001), and 0.86 (*p* < 0.001), respectively. Moreover, the normalized integral errors of the hip, knee, ankle joint moments between the first computer simulation and experiment were 0.09 (0.06), 0.10 (0.05), and 0.40 (0.24), respectively. In Fig. 3c (first computer simulation), the hip, knee, and ankle moment impulses in the sagittal plane were 0.28 ± 0.09, 0.30 ± 0.11, and 0.14 ± 0.06 Nms/kg, respectively. Also, in Fig. 3d (experiment), the hip, knee, and ankle moment impulses in the sagittal plane were 0.23 ± 0.08, 0.36 ± 0.13, and 0.08 ± 0.05 Nms/kg, respectively. Therefore, the hip, knee, and ankle moment impulses obtained from the first computer simulation are 21.7% higher, 16.7% lower, and 75.0% higher respectively, than the experimental values.

**Fig. 4:**
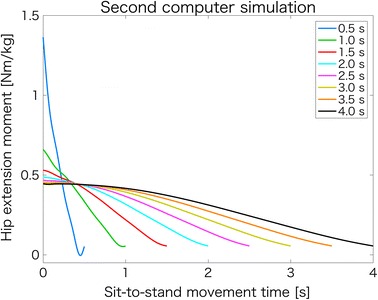
Waveforms of hip extension moments during various sit-to-stand movement times in the second computer simulation. Using 100 trials, sit-to-stand movements during various sit-to-stand movement times were generated for the second computer simulation. Each waveform shows the average of the hip extension moment waveforms of 100 sit-to-stand movements. To make the figure easy to understand, only eight waveforms are shown (i.e., 0.5–4.0 s at intervals of 0.5 s)

Figure 4 presents the hip extension moment waveforms during various sit-to-stand movement times in the second computer simulation. The peak hip extension moment during sit-to-stand movement increased with a decrease in the movement time.

**Fig. 5:**
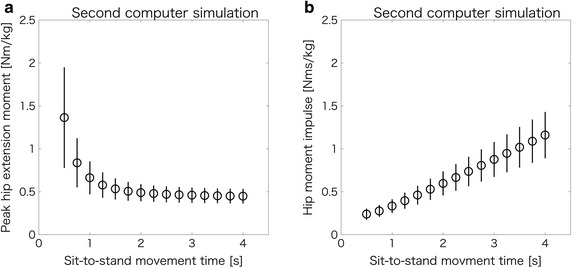
Peak hip extension moments and hip moment impulses in the sagittal plane during various sit-to-stand movement times. **a** Peak hip extension moment decreases with an increase in sit-to-stand movement time. **b** Hip moment impulse in the sagittal plane increases with an increase in sit-to-stand movement time

Figure 5a, b present the peak hip extension moments and hip moment impulses in the sagittal plane during various sit-to-stand movement times in the second computer simulation. In Fig. 5a, the peak hip extension moment during sit-to-stand movement increased exponentially with a decrease in movement time. In Fig. 5b, conversely, the hip moment impulse in the sagittal plane during sit-to-stand movement decreased with a decrease in movement time. Due to multiple comparisons, the significant differences in the peak hip extension moments (Fig. 5a) were found in all combinations (0.5–4.0 s at intervals of 0.25 s, i.e., 15C2 = 105 patterns) of sit-to-stand movement times (*p* < 0.001). Furthermore, significant differences in the hip moment impulses (Fig. 5b) were found in all combinations of sit-to-stand movement times (*p* < 0.001).

## Discussion

### Principal finding

The purpose of this study was to examine the relationship between the movement time and hip moment impulse in the sagittal plane during sit-to-stand movement. We conducted a study that combined an experiment and two computer simulations to achieve our objective. The main finding in this study is that the hip moment impulse in the sagittal plane during sit-to-stand movement decreased with a decrease in the sit-to-stand movement time (Fig. 5b), thereby confirming our hypothesis.

Tateuchi et al. [10] previously reported that daily cumulative hip moments (i.e., the product of the hip moment impulse and steps per day) in the frontal and sagittal planes are risk factors for the progression of hip osteoarthritis. Therefore, although sit-to-stand movement differs from gait, it is important to identify sit-to-stand movement patterns that have a low moment impulse in order to reduce hip joint load. Many previous studies [12–16] have analyzed the hip extension moment during sit-to-stand movements. However, none of them examined the hip moment impulse in the sagittal plane during sit-to-stand movement. Moreover, although it is feasible that a change in sit-to-stand movement time changes the hip moment impulse in the sagittal plane, this effect has not been examined. For these reasons, the main finding of the present study is novel and has important implications for hip osteoarthritis.

However, it is premature for clinicians to advise patients with hip osteoarthritis to stand quickly from a chair to reduce the hip moment impulse in the sagittal plane. Although Tateuchi et al. [10] reported that a high daily cumulative hip moment during gait contributes to narrowing of the hip joint space, other movements were not evaluated. Thus, prior to applying our finding in clinical practice, the effect of the hip moment impulse during other movements (including the sit-to-stand movement) on the progression of hip osteoarthritis should be examined in future cohort studies.

Moreover, although the hip moment impulse during sit-to-stand movement decreased with a decrease in movement time (Fig. 5b), the peak hip extension moment increased exponentially (Fig. 5a). Yoshioka et al. [14] reported that the sum of the peak hip and knee joint moments increased exponentially (especially, inertia component) when the total sit-to-stand movement time was below 2–3 s (i.e., time from seat-off to standing posture was below 1.12–1.68 s). Although the results (Fig. 5a) in the present study do not indicate the sum of the peak hip and knee joint moments, but instead indicate the peak hip extension moments, they do show that the peak hip extension moment increased exponentially when the time from seat-off to standing posture was below approximately 1.12–1.68 s. Indirectly, this agrees with the findings of the previous study [14], and we speculate that the cause is an increase in the inertia component (i.e., an increase in acceleration) in inverse dynamics.

According to several previous studies on knee osteoarthritis, both a high knee adduction moment impulse [35–37] and a high peak knee adduction moment [35, 36, 38] are risk factors for knee osteoarthritis. On the other hand, in a previous study on hip osteoarthritis [10], the peak hip moment was not associated with progression of hip osteoarthritis. However, we consider that an “excessive” peak hip moment may be associated with progression of hip osteoarthritis. To address this question, a detailed examination of risk factors for hip osteoarthritis in future studies is needed.

### Rationale of the experiment and simulation model

First, the sit-to-stand movement time and hip flexion angle at seat-off during normal sit-to-stand movement in the experiment were 0.73 ± 0.16 s and 92.0 ± 6.0°, respectively (Table 1). Yoshioka et al. [14] reported that the mean sit-to-stand movement time was 0.74 ± 0.18 s (calculated from 56% of 1.32 ± 0.33 s) in healthy subjects, and Doorenbosch et al. [12] reported that the hip flexion angle at seat-off was 93.4 ± 8.4° during normal sit-to-stand movement. Therefore, the values in the present experiment align with previous studies [12, 14] and appear to be reasonable.

As reported in previous studies [12–15, 21], normally hip and knee extension moments occur at seat-off and decrease as the subject approaches completion of the sit-to-stand movement. Furthermore, the ankle plantar flexion moment increases as the subject approaches completion of the sit-to-stand movement [21]. The joint moment waveforms during normal sit-to-stand movements in our experiment (Fig. 3b) are corroborated by previous results [12–15, 21].

Moreover, the joint moment waveforms of the hip, knee, and ankle joints during normal sit-to-stand movements in the first computer simulation (Fig. 3a) were also found to be similar to the actual joint moment waveforms (Fig. 3b) observed during normal sit-to-stand movements in the experiment. Indeed, the correlation coefficients of the hip, knee, and ankle joint waveforms between the first computer simulation and the experiment were high (0.99, 0.98, and 0.86, respectively). Also, although the normalized integral error of the ankle joint moment was not low, those values of the hip and knee joint moments were low. Therefore, we speculate that the simulation model of the first computer simulation is quantitatively approximately valid.

Doorenbosch et al. [12] reported that the peak hip extension moment during normal sit-to-stand movement was 0.62 ± 0.12 Nm/kg. In addition, according to another previous study, the peak hip extension moment during sit-to-stand movement using a chair with a seat height of 0.4 m was approximately 1.1 Nm/kg [15]. The peak hip extension moments in our experiment and first computer simulation of normal sit-to-stand movements were 0.71 ± 0.16 and 0.85 ± 0.17 Nm/kg, respectively (Table 1). Therefore, the peak hip extension moment values in our experiment and first computer simulation are comparable to the values reported in the above-mentioned studies [12, 15]. In addition, the hip moment impulse in the first computer simulation (Fig. 3c) was similar to that in the experiment (Fig. 3d). From these findings, we consider that the simulation model created for the first and second computer simulations is valid.

### Limitation

Although we validated the hip joint moment of the simulation model during the normal sit-to-stand movement, we did not evaluate the same during the various sit-to-stand movement times in the second computer simulation. Nevertheless, our results (Fig. 5a) are consistent with another previous study [14]. Although a change in sit-to-stand movement time may cause a change in kinematics [21, 26–29], which may influence the hip moment impulse during sit-to-stand movement, future studies incorporating actual measurements of hip joint moment during various sit-to-to-stand movement times may help to confirm our findings. Furthermore, the subjects who participated in the present study did not have hip osteoarthritis. According to a previous study [16], although there were no distinctive biomechanical alterations in the sagittal or frontal plane kinematics or kinetics in hip osteoarthritis patients compared with control subjects, patients with hip osteoarthritis exhibited a distinct pattern of weight-bearing asymmetry compared with control subjects. Thus, the results of the computer simulation in this study should be compared to results by an experiment of patients with hip osteoarthritis in the future.

## Conclusion

The purpose of this study was to clarify the relationship between the movement time and hip moment impulse in the sagittal plane during sit-to-stand movement. We clarified the relationship using a combination of the experiment and two computer simulations and conclude that rapid sit-to-stand movements could decrease the hip moment impulse in the sagittal plane with a decrease in sit-to-stand movement time. Although it is unknown whether the main finding in the present study can prevent the progression of hip osteoarthritis, the knowledge may help to suggest a sit-to-stand movement pattern with low hip moment impulse in the sagittal plane.
